# Determination of hydrogen exchange and relaxation parameters in PHIP complexes at micromolar concentrations

**DOI:** 10.5194/mr-2-331-2021

**Published:** 2021-05-19

**Authors:** Lisanne Sellies, Ruud L. E. G. Aspers, Marco Tessari

**Affiliations:** Institute for Molecules and Materials, Radboud University, Nijmegen, 6525AJ, the Netherlands

## Abstract

Non-hydrogenative para-hydrogen-induced polarization (PHIP) is a fast, efficient and relatively inexpensive approach to enhance nuclear magnetic resonance (NMR) signals of small molecules in solution. The efficiency of this technique depends on the interplay of NMR relaxation and kinetic processes, which, at high concentrations, can be characterized by selective inversion experiments. However, in the case of dilute solutions this approach is clearly not viable. Here, we present alternative PHIP-based NMR experiments to determine hydrogen and hydride relaxation parameters as well as the rate constants for para-hydrogen association with and dissociation from asymmetric PHIP complexes at micromolar concentrations. Access to these parameters is necessary to understand and improve the PHIP enhancements of (dilute) substrates present in, for instance, biofluids and natural extracts.

## 1 Introduction

**Figure 1:**
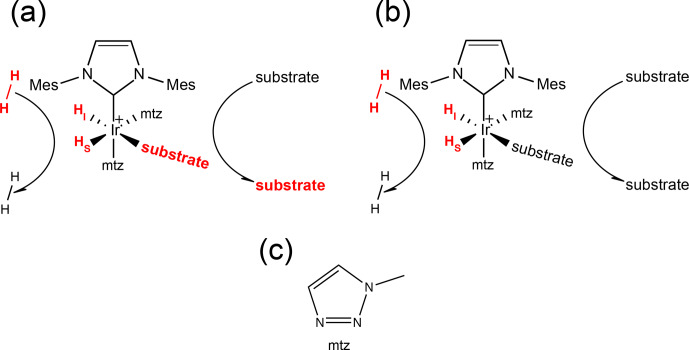
**(a)** Schematic representation of the SABRE experiment at a low magnetic field: spontaneous transfer of spin order from the hydrides originating from p-H ${}_{2}$ to the substrate nuclear spins occurs via the scalar coupling network within the transient complex [Ir(IMes)(H) ${}_{2}$ (substrate)(mtz) ${}_{2}$ ]Cl. The subsequent dissociation of the substrate produces hyperpolarized molecules in solution that can be detected by NMR with enhanced sensitivity. SABRE hyperpolarization has been demonstrated for different classes of compounds, e.g., nitrogen (Adams et al., 2009) and sulfur (Shchepin et al., 2016) heteroaromatic compounds, nitriles (Mewis et al., 2015), amines (Iali et al., 2018), Schiff bases (Logan et al., 2016) and diazirines (Theis et al., 2016). **(b)** Schematic representation of PHIP at high magnetic field: formation of the asymmetric complex [Ir(IMes)(H) ${}_{2}$ (substrate)(mtz) ${}_{2}$ ]Cl due to the reversible association of p-H ${}_{2}$ and substrates produces longitudinal spin order of the hydrides, which can be revealed by NMR signals that are enhanced by up to 3 orders of magnitude compared to thermal measurements on a conventional high-field spectrometer. **(c)** Structure of the mtz co-substrate (1-methyl-1,2,3-triazole).

The intrinsically low sensitivity of magnetic resonance techniques is a strong limitation to their application in fields such as chemical analysis, metabolic imaging and biomarker identification. Several hyperpolarization methods have been developed to overcome this issue, including dynamic nuclear polarization (Ardenkjær-Larsen et al., 2003), spin exchange optical pumping (Walker and Happer, 1997) and para-hydrogen-induced polarization (PHIP) (Bowers and Weitekamp, 1987; Pravica and Weitekamp, 1988). Particularly, PHIP has grown into a versatile technique since the recent discovery of non-hydrogenative routes to achieve nuclear spin hyperpolarization (Adams et al., 2009). Figure 1 sketches the core of a typical non-hydrogenative PHIP machinery, based on the reversible association of para-hydrogen (p-H ${}_{2}$ ) and substrates with an iridium catalyst. We have previously demonstrated that a large excess of a suitable metal ligand (e.g., 1-methyl-1,2,3-triazole, mtz), referred to as “co-substrate” in the following, is necessary to preserve the efficiency of non-hydrogenative PHIP when the substrate under investigation is highly dilute (Eshuis et al., 2014, 2015).

In Fig. 1a, the signal amplification by reversible exchange (SABRE) (Adams et al., 2009) technique is sketched: at low magnetic field, the scalar coupling network within the transient complex allows the spontaneous transfer of spin order from the hydrides (derived from p-H ${}_{2}$ binding) to the nuclear spins of the substrate molecules. Subsequent complex dissociation releases hyperpolarized substrate molecules in solution, which can be detected with nuclear magnetic resonance (NMR) with sensitivity enhanced by several orders of magnitude (Theis et al., 2015; Rayner et al., 2017; Rayner and Duckett, 2018; Iali et al., 2019; Gemeinhardt et al., 2020).

Alternatively, the transient complex itself offers the possibility of investigating the substrates bound to the catalyst (see Fig. 1b). We have previously demonstrated that such an asymmetric complex is an ideal NMR chemosensor (Hermkens et al., 2016; Sellies et al., 2019): molecules capable of associating with the PHIP catalyst can be probed by a pair of hydride signals enhanced by ca. 3 orders of magnitude with respect to thermal NMR measured at 500 MHz. This allows the detection and quantification of substrates present at sub-micromolar concentrations in complex mixtures (Eshuis et al., 2015), such as biofluids (Reile et al., 2016; Sellies et al., 2019) and natural extracts (Hermkens et al., 2016, 2018).

The NMR signal enhancements obtained by these reversible PHIP techniques result from a complex interplay of substrate exchange, para-hydrogen exchange and relaxation processes (Barskiy et al., 2016; Stanbury et al., 2019). For instance, at a high field, the singlet state of para-hydrogen associating with an asymmetric complex rapidly turns into hydrides' longitudinal spin order, which can be converted into enhanced hydride magnetization by a SEPP pulse sequence (Sengstschmid et al., 1996). The resulting hydrides' signal enhancement depends, therefore, amongst others, on the para-hydrogen lifetime in solution, the rates of para-hydrogen association/dissociation with/from the complex, and the NMR relaxation of the longitudinal spin order in the complex. Gaining access to these parameters is crucial in order to rationalize the observed variations in hydrides' NMR signal enhancement for different substrates (Sellies et al., 2019) or to optimally tune the chemosensing system (e.g., select the best co-substrate molecule) for specific substrates and/or experimental applications. Since some of these parameters, e.g., the hydrogen dissociation rate constant, strongly depend on the concentration of ligands in solution (Cowley et al., 2011; Appleby et al., 2015), it is important to measure them in the same dilute conditions normally encountered in PHIP NMR.

The association and dissociation rates of hydrogen and substrates with/from the iridium complex can be determined using exchange NMR spectroscopy (EXSY), in which, for instance, the longitudinal magnetization of one of the two exchanging forms is selectively inverted and probed together with its exchange product after different durations of a mixing time (Cowley et al., 2011; Appleby et al., 2015). These experiments are typically performed at high concentrations of substrate and catalyst, making them unsuitable for characterizing low-concentrated complexes. Instead, the hyperpolarization provided by para-hydrogen can be exploited, as we previously demonstrated for the measurement of the substrate dissociation rate from asymmetric complexes (Hermkens et al., 2017).

Here, we present two PHIP-NMR experiments to characterize the hydrides' dynamics in these asymmetric complexes at low concentration. The analysis of the resulting NMR data provides not only the hydrogen exchange rates in the low µ M regime, but also the hydrides' relaxation parameters. We illustrate this approach for the asymmetric complex formed upon binding of the substrate isoquinoline (IQ) to the Ir–IMes catalyst in the presence of an excess of mtz as co-substrate.

## 2 Material and methods

### 2.1 Chemicals

Complex precursor [IrCl(COD)(IMes)] (IMes = 1,3-bis(2,4,6-trimethylphenyl)imidazole-2-ylidene; COD = cyclooctadiene) and co-substrate mtz were synthesized according to published methods (Kelly et al., 2008; Seefeld et al., 2008). Isoquinoline and methanol-d ${}_{4}$ were purchased from Sigma-Aldrich and used as supplied. Para-hydrogen (p-H ${}_{2}$ ) was produced with an in-house-designed generator (Cryoworld B.V., the Netherlands) consisting of a 2 L vessel embedded in a liquid nitrogen bath. Normal hydrogen (purity 5.0) at 40 bar was cooled down to 77 K in the presence of 100 mL of 4–8 MESH charcoal (Sigma-Aldrich). The resulting 51 % p-H ${}_{2}$ was transferred into an aluminum cylinder (Nitrous Oxides Systems, Holley Performance Products, USA) (Feng et al., 2012) and connected to a set-up for gas–liquid reactions (Eshuis et al., 2015), as sketched in Appendix A.

### 2.2 Sample preparation and set-up

[IrCl(COD)(IMes)], mtz and isoquinoline were mixed to final concentrations of 0.8 mM, 15 mM and 50 µ M in methanol-d ${}_{4}$ , respectively. The solution was transferred into a 5 mm quick pressure valve (QPV) NMR tube (Wilmad-LabGlass). This tube was sealed with an in-house-built headpiece to which three PEEK tube lines are connected (see Appendix A). Nitrogen gas was passed through the solution to remove dissolved oxygen, after which [IrCl(COD)(IMes)] was hydrogenated (activated) by bubbling p-H ${}_{2}$ through the solution for 1.5 s every 2 min for approximately 30 min.

### 2.3 p-H ${}_{2}$ supply

At the beginning of each transient of an NMR experiment, the sample tube was depressurized to 4 bar through a vent line (250 ms), after which p-H ${}_{2}$ at 5 bar pressure was supplied for 1.5 s through a line ending at the bottom of the NMR tube. Back pressure was applied to quickly stop the bubbling (250 ms), followed by a recovery delay of 500 ms prior to the NMR pulse sequence. The vent-, bubble-, and back-pressure delays are spectrometer-controlled through solenoid valves connected to the console (see Appendix A).

### 2.4 NMR experiments

All NMR experiments were performed at 25 ${}^{\circ }$ C on an Agilent Unity INOVA spectrometer operating at 500 MHz ${}^{1}$ H resonance frequency using a cryo-cooled HCN triple-resonance probe equipped with z-pulsed field gradients.

The datasets employed in this study consist of series of 18 1D PHIP-NMR spectra acquired with variable exchange/relaxation periods Δ ranging between 100 ms and 5 s. The transmitter offset was placed at - 11.35 ppm, and the spectral region between - 31.36 and 8.66 ppm was acquired for 0.5 s. Four or eight transients were recorded per each Δ duration corresponding to an experimental time of 10 or 20 min for a complete series. In order to avoid variations in the level of p-H ${}_{2}$ in solution, the time interval between two successive bubbling periods (at the beginning of each transient) was kept constant, independent of the duration of Δ .

The decay rate of p-H ${}_{2}$ in solution was determined in a separate experiment by acquiring a series of 48 single-scan 1D PHIP-NMR signals of the high-field hydride in the asymmetric complex formed by isoquinoline, mtz, and the iridium catalyst. After bubbling p-H ${}_{2}$ in solution at the beginning of the experiment, all spectra were acquired (one per second) without refreshing p-H ${}_{2}$ during the measurement. As the p-H ${}_{2}$ concentration in solution decreases, the PHIP enhancement of the NMR hydride signal drops. The rate constant of the conversion of para-enriched H ${}_{2}$ to thermal hydrogen was obtained by the exponential fit of the signal integral versus time.

All datasets were processed with nmrPipe (Delaglio et al., 1995) and analyzed with iNMR (Balacco and Marino, 2005) using 90 ${}^{\circ }$ shifted squared sine-bell apodization, prior to zero filling to 128k complex points, and Fourier transformation. The fitting of NMR signal integrals versus exchange/relaxation time Δ was performed using in-house-written routines implemented in Octave (Eaton et al., 2009).

## 3 Theory

### 3.1 PHIP-NMR pulse sequences for hydrogen kinetics/relaxation

At high magnetic field the two hydrides of asymmetric Ir–IMes complexes (see Fig. 1) are not chemically equivalent, which, due to the distribution of p-H ${}_{2}$ association in time, causes rapid conversion of the singlet state to longitudinal spin order (Buljubasich et al., 2013). We have previously demonstrated that this spin order can be converted into enhanced magnetization, allowing the NMR detection of hydride signals down to sub-micromolar complex concentrations (Eshuis et al., 2015; Sellies et al., 2019). This sensitivity increase can also be used to study the exchange of p-H ${}_{2}$ in the iridium catalyst as well as the NMR relaxation of the hydrides and p-H ${}_{2}$ in solution.

**Figure 2:**
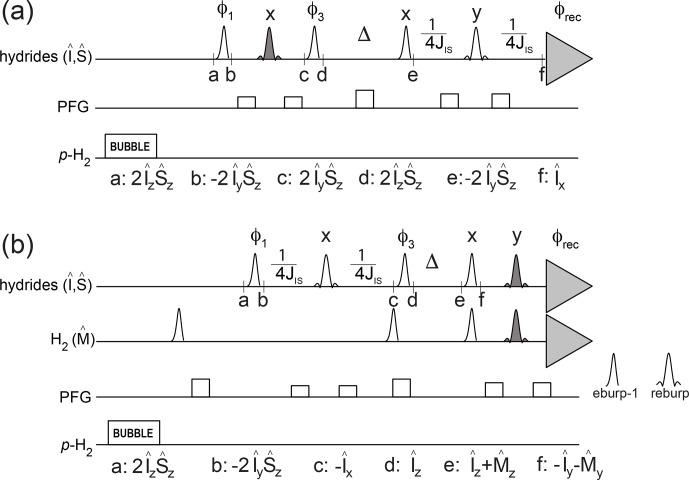
Pulse sequences to measure relaxation and kinetic parameters for hydrides in asymmetric complexes. The transmitter offset is placed at - 11.35 ppm, and the spectral region between - 31.36 and 8.66 ppm is acquired. Open-shaped profiles indicate selective off-resonance pulses *eburp-1* (for excitation of the high-field hydride, 500 Hz bandwidth) or *reburp* (for refocusing, 2000 Hz bandwidth). Filled *reburp* pulses cover a smaller bandwidth (500 Hz) for the hydrogen and/or the high-field hydride resonance. Individual scans are stored separately and recombined during processing so that different pathways can be selected. $J_{\mathrm{IS}}$ indicates the inter-hydride scalar coupling constant (8.2 Hz). **(a)** Pulse scheme to measure the decay rate of hydrides' longitudinal spin order as well as association of p-H ${}_{2}$ with the iridium complex. The following phase cycle is employed: $\varphi_{1}=x,-x$ ; $\varphi_{3}=x,x,-x,-x$ ; $\varphi_{\mathrm{rec}}=x$ . **(b)** Pulse scheme to measure the decay rate of hydrides' longitudinal magnetization as well as hydride dissociation from the complex. The following phase cycle is employed: $\varphi_{1}=x,-x$ ; $\varphi_{3}=y,y,-y,-y$ ; $\varphi_{\mathrm{rec}}=x,-x,-x,x$ . H ${}_{2}$ longitudinal magnetization is suppressed at the start of the pulse sequence by selective 90 ${}^{\circ }$ pulses after the bubbling period and at the beginning of the relaxation period Δ . The final excitation/spin echo before acquisition selectively excites/refocuses both the magnetization from H ${}_{2}$ and from the high-field hydride. In other words, the last excitation/refocusing pulses consist of the superposition of two *eburp-1*/*reburp* pulses, for the selective detection of the high-field hydride as well as the hydrogen resonances.

The pulse schemes in Fig. 2 make use of PHIP NMR to quantitatively characterize these kinetic and relaxation parameters for asymmetric complexes at low micromolar concentration, i.e., the conditions under which p-H ${}_{2}$ hyperpolarization is typically used for the detection of dilute substrates. The relevant spin operators at specified time points in the pulse schemes are indicated.

The experiment sketched in Fig. 2a can monitor p-H ${}_{2}$ association as well as the decay of the hydrides' spin order as a function of the delay time Δ . The first spin echo (between time points a and d ) allows implementation of a phase cycle to separate the hydride signals produced by p-H ${}_{2}$ association with the complex during the bubbling period from those resulting from p-H ${}_{2}$ association during the exchange/relaxation period Δ . After the time Δ the spin order is converted to antiphase magnetization, refocused and acquired. By storing each individual scan separately, it is then possible to monitor either the hydride decay or the association of p-H ${}_{2}$ with the complex during Δ by taking different combinations of the acquired signals (e.g., multiplying some scans by - 1 before adding them together, corresponding to a 180 ${}^{\circ }$ phase shift of the receiver).

The pulse scheme in Fig. 2b was used to monitor the decay of the hydrides' longitudinal magnetization as well as the hydrides' dissociation to produce hyperpolarized hydrogen in solution as a function of the mixing time Δ . After the bubbling period, the hydrides' spin order is converted to antiphase magnetization, refocused and stored as longitudinal magnetization during the exchange/relaxation time Δ . After this time, the remaining hydrides' magnetization as well as the transferred hydrogen magnetization are excited and acquired simultaneously, in the same spectrum.

### 3.2 Spin dynamics

The evolution of the hydrides' magnetization/spin order during the relaxation/exchange time Δ is described by the equations below.

1ddt2I^zS^zB2I^zS^zF=-kdiss∗+ρhydrso-kass∗-kdiss∗kass∗+ρpH22I^zS^zB2I^zS^zF2ddtI^zBI^zF=-kdiss∗+ρhydr-kass∗-kdiss∗kass∗+ρH2I^zB-I^eqBI^zF-I^eqF

Equation (1) describes the kinetics and NMR relaxation of the longitudinal spin order of hydrides (index “B”) and of free hydrogen (index “F”). Here, $k_{\mathrm{diss}}^{*}$ and $k_{\mathrm{ass}}^{*}$ represent the dissociation and association rate constants of hydrogen from/with the asymmetric Ir–IMes complexes, $\rho_{\mathrm{hydr}}^{\mathrm{so}}$ the relaxation of hydrides' longitudinal spin order in these complexes and $\rho_{pH_{2}}$ the rate of thermalization of para-enriched H ${}_{2}$ . The asterisk marking the kinetic rate constants indicates that they most likely result from multi-step processes, and their physical interpretation strictly depends on the hypothesized mechanism. Note that the (unobservable) term $(2{\hat{I}}_{z}{\hat{S}}_{z}{)}^{F}$ refers here to both p-H ${}_{2}$ as well as the longitudinal spin order of free hydrogen (Barskiy et al., 2019). Analogously, Eq. (2) describes the dynamics of the longitudinal magnetization of hydrides and free hydrogen in solution; in this case the kinetic processes involved are identical, while $\rho_{\mathrm{hydr}}$ and $\rho_{H_{2}}$ refer to the spin–lattice relaxation rates of the hydrides and of free hydrogen, respectively. Note that in the presence of cross-correlated relaxation mechanisms, the dynamics of $\langle {\hat{I}}_{z}\rangle$ and $\langle 2{\hat{I}}_{z}{\hat{S}}_{z}\rangle$ are coupled and are not described by two independent equations (Eqs. 1 and 2). In the present case, however, such cross terms are likely to be negligible, and their effect has not been considered.

It should be mentioned that in principle substrate and co-substrate dissociation might also contribute to the decay of the hydride magnetization and spin order ( $\langle ({\hat{I}}_{z}{)}^{B}\rangle$ and ( $\langle 2{\hat{I}}_{z}{\hat{S}}_{z}{)}^{B}\rangle$ ). These additional processes can be easily followed as they produce a magnetization transfer from the high-field to the low-field hydride or to the hydrides of the symmetric complex ([Ir(IMes)(H) ${}_{2}$ (mtz) ${}_{3}$ ] ${}^{+}$ ). However, in the present case such transfer was not observed, indicating that these exchange processes occur at a significantly lower rate than the hydrides' relaxation for the complex here investigated. Therefore, the decay rates of the hydrides ( $\rho_{\mathrm{hydr}}$ and $\rho_{\mathrm{hydr}}^{\mathrm{so}}$ ) determined in this work correspond to NMR relaxation parameters to a very good approximation.

The solution of Eq. (1) refers here only to the dynamics of the hydrides' longitudinal spin order, as the corresponding term for free hydrogen is not observable.

3 $$\begin{array}{rl} & \langle {\left(2{\hat{I}}_{z}{\hat{S}}_{z}\right)}^{B}\rangle \left(\Delta \right)=\frac{e^{-\left({\bar{\rho }}^{\mathrm{so}}+\bar{k}\right)\Delta }}{1+\frac{\left({\bar{k}}^{2}-\Delta k^{2}\right)}{{\left(\Delta \rho^{\mathrm{so}}+\Delta k+\varepsilon^{\mathrm{so}}\right)}^{2}}}\\ & \times \left\{\left(e^{-\varepsilon^{\mathrm{so}}\Delta }+\frac{\left({\bar{k}}^{2}-\Delta k^{2}\right)}{{\left(\Delta \rho^{\mathrm{so}}+\Delta k+\varepsilon^{\mathrm{so}}\right)}^{2}}e^{\varepsilon^{\mathrm{so}}\Delta }\right)\langle {\left(2{\hat{I}}_{z}{\hat{S}}_{z}\right)}^{B}\rangle (0)\right.\\ & \left.+\frac{\left(\bar{k}-\Delta k\right)}{\left(\Delta \rho^{\mathrm{so}}+\Delta k+\varepsilon^{\mathrm{so}}\right)}\left(e^{\varepsilon^{\mathrm{so}}\Delta }-e^{-\varepsilon^{\mathrm{so}}\Delta }\right)\langle {\left(2{\hat{I}}_{z}{\hat{S}}_{z}\right)}^{F}\rangle (0)\right\} \end{array}$$

$$\begin{array}{rl} & {\bar{\rho }}^{\mathrm{so}}=\frac{\rho_{\mathrm{hydr}}^{\mathrm{so}}+\rho_{pH_{2}}}{2},\;\Delta \rho^{\mathrm{so}}=\frac{\rho_{\mathrm{hydr}}^{\mathrm{so}}-\rho_{pH_{2}}}{2},\\ & \bar{k}=\frac{k_{\mathrm{diss}}^{*}+k_{\mathrm{ass}}^{*}}{2},\;\Delta k=\frac{k_{\mathrm{diss}}^{*}-k_{\mathrm{ass}}^{*}}{2},\\ & \varepsilon^{\mathrm{so}}=\sqrt{{\left(\Delta \rho^{\mathrm{so}}+\Delta k\right)}^{2}+{\bar{k}}^{2}-\Delta k^{2}} \end{array}$$

By combining individual scans with coefficients {1,-1,-1,1} , it is possible to select the signal resulting from the hydrides associated with the complex during the bubbling period while discarding the contribution originating from the association of p-H ${}_{2}$ during the time Δ . In this case, only the first term of Eq. (3) should be considered:

4 $$\begin{array}{rl} & \langle {\left(2{\hat{I}}_{z}{\hat{S}}_{z}\right)}^{B}\rangle \left(\Delta \right)=\frac{e^{-\left({\bar{\rho }}^{\mathrm{so}}+\bar{k}\right)\Delta }}{1+\frac{\left({\bar{k}}^{2}-\Delta k^{2}\right)}{{\left(\Delta \rho^{\mathrm{so}}+\Delta k+\varepsilon^{\mathrm{so}}\right)}^{2}}}\\ & \times \left(e^{-\varepsilon^{\mathrm{so}}\Delta }+\frac{\left({\bar{k}}^{2}-\Delta k^{2}\right)}{{\left(\Delta \rho^{\mathrm{so}}+\Delta k+\varepsilon^{\mathrm{so}}\right)}^{2}}e^{\varepsilon^{\mathrm{so}}\Delta }\right)\langle {\left(2{\hat{I}}_{z}{\hat{S}}_{z}\right)}^{B}\rangle (0). \end{array}$$

Equation (4) describes the decay of the hydrides' spin order due to NMR relaxation and dissociation of the hydrides from the complex. If, alternatively, individual scans are combined with coefficients {1,1,1,1} , only the signal originating from p-H ${}_{2}$ associating during the relaxation time Δ is observed. The time evolution of the measured signal is described by the second term of Eq. (3):

5 $$\begin{array}{rl} & \langle {\left(2{\hat{I}}_{z}{\hat{S}}_{z}\right)}^{B}\rangle \left(\Delta \right)=\frac{e^{-\left({\bar{\rho }}^{\mathrm{so}}+\bar{k}\right)\Delta }}{1+\frac{\left({\bar{k}}^{2}-\Delta k^{2}\right)}{{\left(\Delta \rho^{\mathrm{so}}+\Delta k+\varepsilon^{\mathrm{so}}\right)}^{2}}}\\ & \times \frac{\left(\bar{k}-\Delta k\right)}{\left(\Delta \rho^{\mathrm{so}}+\Delta k+\varepsilon^{\mathrm{so}}\right)}\left(e^{\varepsilon^{\mathrm{so}}\Delta }-e^{-\varepsilon^{\mathrm{so}}\Delta }\right)\langle {\left(2{\hat{I}}_{z}{\hat{S}}_{z}\right)}^{F}\rangle (0). \end{array}$$

The time evolution of the longitudinal magnetization of free hydrogen and hydrides during the period Δ is given by the solution of Eq. (2):

6I^zBΔ=e-ρ‾+k‾Δ1+k‾2-Δk2Δρ+Δk+ε2×e-εΔ+k‾2-Δk2Δρ+Δk+ε2eεΔI^zB(0)+k‾-ΔkΔρ+Δk+εeεΔ-e-εΔI^zF(0),7I^zFΔ=e-ρ‾+k‾Δ1+k‾2-Δk2Δρ+Δk+ε2×k‾+ΔkΔρ+Δk+εeεΔ-e-εΔI^zB(0)+eεΔ+k‾2-Δk2Δρ+Δk+ε2e-εΔI^zF(0),

$$\begin{array}{rl} & \bar{\rho }=\frac{\rho_{\mathrm{hydr}}+\rho_{H_{2}}}{2},\;\Delta \rho =\frac{\rho_{\mathrm{hydr}}-\rho_{H_{2}}}{2},\\ & \bar{k}=\frac{k_{\mathrm{diss}}^{*}+k_{\mathrm{ass}}^{*}}{2},\;\Delta k=\frac{k_{\mathrm{diss}}^{*}-k_{\mathrm{ass}}^{*}}{2},\\ & \varepsilon =\sqrt{{\left(\Delta \rho +\Delta k\right)}^{2}+{\bar{k}}^{2}-\Delta k^{2}}. \end{array}$$

In this case, both the bound form (i.e., the hydrides) and free hydrogen are observable. Since the phase cycle selects the signal originating from the hydrides associated with the complex during the bubbling period, while removing the contribution due to free hydrogen associating with the complex during Δ , the solution to Eq. (2) takes this form:

8I^zBΔ=e-ρ‾+k‾Δ1+k‾2-Δk2Δρ+Δk+ε2×e-εΔ+k‾2-Δk2Δρ+Δk+ε2eεΔI^zB(0),9I^zFΔ=e-ρ‾+k‾Δ1+k‾2-Δk2Δρ+Δk+ε2k‾+ΔkΔρ+Δk+ε×eεΔ-e-εΔI^zB(0).

Note that in both expressions the contributions of thermal magnetization for hydrogen and hydrides are suppressed by the phase cycle.

**Figure 3:**
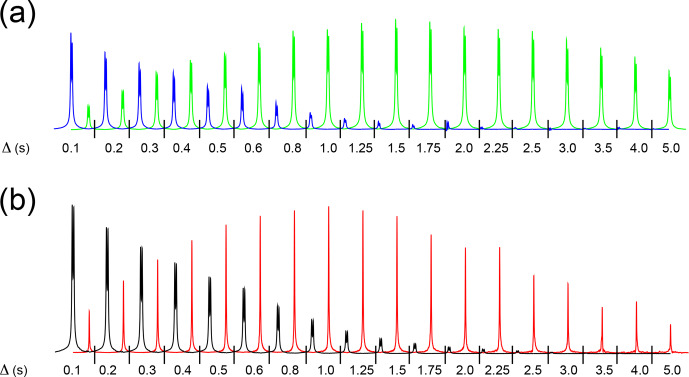
**(a)** High-field hydride signals obtained with the pulse sequence sketched in Fig. 2a, plotted as a function of the exchange/relaxation period Δ . The experiment monitors the decay of the longitudinal spin order of the hydrides or the association of p-H ${}_{2}$ with the complex during Δ . By taking different combinations of four FIDs as reported in Sect. 3.2, the decay (blue) or the buildup (green) of the hydride signal is obtained. The total experimental duration was 10 min. **(b)** Decay of the high-field hydride (black) and buildup of free H ${}_{2}$ (red) signals obtained with the pulse sequence sketched in Fig. 2b, plotted as a function of the exchange/relaxation period Δ . The experiment monitors the decay of the longitudinal magnetization of the hydrides or the hydrides' dissociation during Δ . Eight scans were measured for each spectrum, for a total experimental duration of 20 min. Both experiments were recorded using a solution of 0.8 mM metal complex, 15 mM mtz, 50 µ M isoquinoline dissolved in methanol-d ${}_{4}$ in the presence of 5 bar 51 % enriched p-H ${}_{2}$ at 25 ${}^{\circ }$ C.

## 4 Results and discussion

Previous studies (Cowley et al., 2011; Appleby et al., 2015) have clearly demonstrated the influence of substrates and catalyst concentrations on the hydrogen dissociation rate and, as a consequence, on the signal enhancement attainable via PHIP/SABRE. Therefore, in order to understand and improve the efficiency of PHIP for substrates in dilute asymmetric complexes, it is important to determine the relevant kinetic parameters at low concentrations. In the present study, isoquinoline at 50 µ M concentration was used as a substrate together with mtz as a co-substrate and iridium–IMes as a metal complex. This combination of co-substrate and metal complex was previously utilized to detect dilute substrates in complex mixtures (Eshuis et al., 2014, 2015; Bordonali et al., 2019). The following experimental data, displayed in Fig. 3, were recorded using the two NMR experiments described above:

- the decay of hydride longitudinal spin order as a function of the relaxation delay Δ (Fig. 3a, blue),
- the buildup of hydride spin order due to association of p-H ${}_{2}$ with the complex as a function of the exchange delay Δ (Fig. 3a, green),
- the decay of hydride magnetization as a function of the relaxation delay Δ (Fig. 3b, black), and
- the buildup of H ${}_{2}$ magnetization resulting from hydride dissociation as a function of the exchange delay Δ (Fig 3b, red).

The data were fitted simultaneously with the corresponding equations by optimization of the following parameters: $\rho_{\mathrm{hydr}}$ , $\rho_{\mathrm{hydr}}^{\mathrm{so}}$ , $\rho_{H_{2}}$ , $\rho_{pH_{2}}$ , $k_{\mathrm{diss}}^{*}$ , $k_{\mathrm{ass}}^{*}$ , $\langle (2{\hat{I}}_{z}{\hat{S}}_{z}{)}^{F}\rangle (0)$ , $\langle (2{\hat{I}}_{z}{\hat{S}}_{z}{)}^{B}\rangle (0)$ , and $\langle ({\hat{I}}_{z}{)}^{B}\rangle (0)$ .

**Figure 4:**
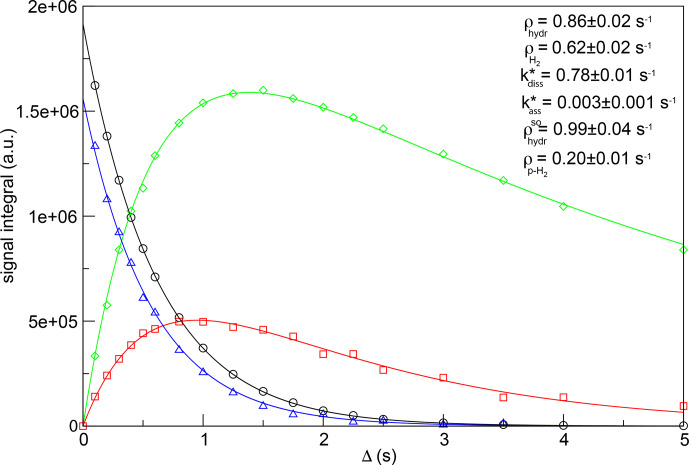
Simultaneous fit as a function of the relaxation period Δ of the signal integrals of hydrides and H ${}_{2}$ derived from the experimental data of Fig. 3 to determine the longitudinal magnetization decay rate (black circles), hydride dissociation rate (red squares), p-H ${}_{2}$ association rate (green diamonds) and longitudinal spin order decay rate (blue triangles) in the asymmetric complex [Ir(IMes)(H) ${}_{2}$ (IQ)(mtz) ${}_{2}$ ]Cl. The values of the hydrogen dissociation and association rate constants and the hydrogen/hydrides' relaxation rates are indicated. The experimental data and the fitting curves have been rescaled to the same number of scans for this plot.

Figure 4 displays the fitting of the experimental data, together with the optimized values of kinetic constants and relaxation rates. Errors associated with the fitting parameters were estimated by the jackknife method (Caceci, 1989). Note that the fitting was performed without constraining any parameter. Nevertheless, the obtained value for the recovery rate for H ${}_{2}$ (0.62 ± 0.02 s ${}^{-1}$ ) is in good agreement with the one measured for the same sample performing a saturation-recovery experiment (0.603 ± 0.003 s ${}^{-1}$ ). Similarly, the experimental value of the decay rate for p-H ${}_{2}$ (0.226 ± 0.003 s ${}^{-1}$ ) is consistent with the result obtained from this fitting (0.20 ± 0.01 s ${}^{-1}$ ).

As previously stated, the values of hydrogen dissociation/association rates reflect a multi-step process and, therefore, detailed knowledge of the kinetic mechanism is necessary for their interpretation. However, the value of 0.78 s ${}^{-1}$ here determined for the hydrogen dissociation rate constant indicates a relatively long lifetime of the [Ir(IMes)(H) ${}_{2}$ (IQ)(mtz) ${}_{2}$ ] ${}^{+}$ asymmetric complex. This time stability seems to be a common feature of asymmetric complexes involving mtz as a co-substrate, a positive aspect for PHIP chemosensing applications in complex mixture analysis, in which high-resolution 2D NMR spectra are necessary to resolve highly crowded regions. Thanks to the stability of these mtz complexes, we have been able to acquire well-resolved signals of low-concentrated metabolites in urine extracts, using 2D PHIP-NMR spectra with evolution times exceeding 500 ms (Sellies et al., 2019). However, it should be mentioned that such high resolution comes at the cost of lower PHIP enhancements, due to a reduced p-H ${}_{2}$ refreshment rate. Therefore, a different co-substrate should be favored if maximal sensitivity is required.

## 5 Conclusions

We have presented an efficient approach for the experimental determination of the relaxation rates and kinetic parameters for p-H ${}_{2}$ association/dissociation in asymmetric PHIP complexes. The proposed PHIP-NMR experiments were tested for the substrate isoquinoline in combination with mtz as co-substrate and Ir–IMes as metal complex. We have thereby demonstrated that, thanks to the signal enhancement provided by PHIP, these NMR experiments can be efficiently employed even at low micromolar complex concentrations. Together with our recently published PHIP experiments to probe the substrate kinetics and relaxation rates (Hermkens et al., 2017), detailed experimental characterization of the parameters underlying PHIP signal enhancements can now be obtained for substrates at low µ M concentrations. We believe that access to these parameters might help in understanding the variations in PHIP enhancements for different substrates. Furthermore, it could guide a rational design of new PHIP catalysts as well as the choice for the optimal co-substrate for the desired application.

## Figures and Tables

**Figure A1:**
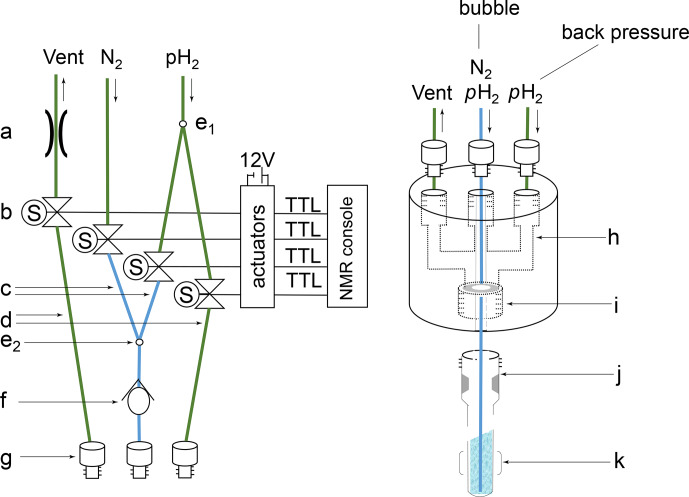
Schematic representation of the gas–liquid reaction set-up. Left: **(a)** variable pressure relief valve, set to 4 bar; **(b)** solenoid valves controlled via TTL lines powered by an external 12 V source and timed via trigger commands in the pulse sequence taking care of a controlled supply of nitrogen and para-hydrogen as described in Sect. 2.2 and 2.3; **(c)** $1/16^{\prime \prime }$ O.D. PEEK tubing with 0.010 ${}^{\prime \prime }$ I.D. (blue); **(d)** $1/16^{\prime \prime }$ O.D. PEEK tubing with 0.030 ${}^{\prime \prime }$ I.D. (green); **(e)** Y splitter: (**e** ${}_{1}$ ) splitting 5 bar p-H ${}_{2}$ source into a “bubble” and a “back pressure” line, (**e** ${}_{2}$ ) combining 5 bar N ${}_{2}$ and p-H ${}_{2}$ sources into a joined bubble line; **(f)** check valve; **(g)** tube fitting UNF 10-32 connectors. Right: **(h)** headpiece connecting the PEEK tubing holding the 7 ${}^{\prime \prime }$ thin wall QPV NMR tube; **(i)** UNF 7/16-20 thread holding the QPV NMR tube – using a 2 mm silicon disc on top of the tube to close off the system; **(j)** Wilmad 7 ${}^{\prime \prime }$ thin wall QPV NMR tube; **(k)** area of detection with the bubble line centered down to the bottom of the NMR tube.

**Figure B1:**
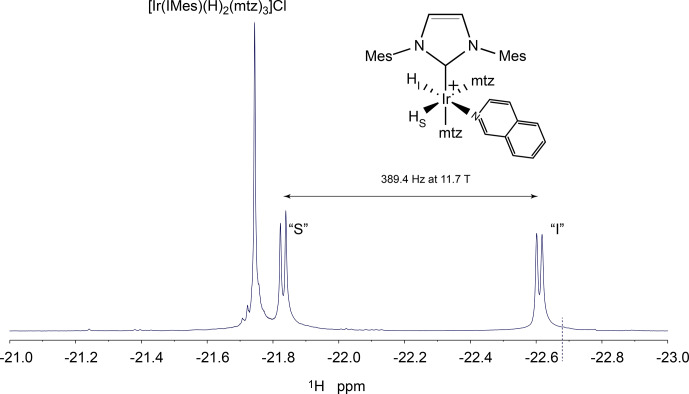
PHIP-NMR spectrum of the hydride region of a solution of isoquinoline 50 µ M, iridium–IMes complex 0.8 mM, mtz 15 mM in methanol-d ${}_{4}$ in the presence of 5 bar 51 % enriched p-H ${}_{2}$ . The experiment was recorded with 16 scans at 25 ${}^{\circ }$ C using a SEPP pulse sequence on an Agilent Unity Inova NMR spectrometer operating at 500 MHz proton resonance frequency. The structure of the asymmetric complex formed by mtz and isoquinoline is indicated together with the assignment of the two hydrides. The frequency difference between the two hydride signals at 500 MHz is reported. The dotted line in proximity to the signal of hydride “I” indicates the selective excitation frequency for the hydrides employed in the kinetics experiments described in Sect. 3.1.

## Data Availability

Experimental data, processed data and nmrPipe processing scripts are openly available from the DANS EASY archive at https://doi.org/10.17026/dans-x6c-zvrp (Tessari et al., 2021).
